# Hemophagocytic syndrome in a cat

**DOI:** 10.1177/2055116918795023

**Published:** 2018-08-27

**Authors:** Ashley R Wilkinson, Susan V Carr, Shawna L Klahn, Nikolaos G Dervisis, Cory R Hanks

**Affiliations:** Virginia-Maryland College of Veterinary Medicine, Blacksburg, VA, USA

**Keywords:** Anemia, bone marrow, immune-mediated diseases, cardiomyopathy, hemophagocytic syndrome

## Abstract

**Case summary:**

A 12-year-old male castrated domestic shorthair cat was evaluated for a 10 month history of weight loss. Thin body condition and a grade II/VI systolic parasternal heart murmur was noted during examination. Moderate-to-severe anemia and intermittent thrombocytopenia were identified on serial complete blood counts. Antibodies against feline immunodeficiency virus (FIV) were detected, but vaccination for FIV occurred previously. Echocardiography revealed biatrial and biventricular enlargement, left ventricular hypertrophy and pericardial effusion. Splenomegaly was present on abdominal ultrasound and cytological evaluation revealed macrophagic infiltration with erythrophagocytosis. Cytological evaluation of the bone marrow revealed similar findings. Histopathology of the spleen confirmed hemophagocytosis with no evidence of malignancy. A presumptive diagnosis of hemophagocytic syndrome was made. PCR testing for FIV on the splenic tissue was negative. The cat was treated with lomustine. Disease progression occurred approximately 6 months after diagnosis and the cat was euthanized.

**Relevance and novel information:**

To our knowledge, this is one of the few reports describing the diagnosis of hemophagocytic syndrome in a cat.

## Case description

A 12-year-old male castrated domestic shorthair cat was referred to the Virginia-Maryland College of Veterinary Medicine Veterinary Teaching Hospital (VTH) for weight loss of 10 months’ duration and anemia of 3 weeks’ duration. Abnormalities identified by the primary veterinarian 3 weeks prior to admission included a thin body condition and a grade II/VI holosystolic heart murmur. Serum biochemical profile and total thyroxine were within normal limits. Complete blood count (CBC) revealed a normocytic, normochromic, regenerative anemia (hematocrit [HCT] 17.2%, reference interval [RI] 30.3–52.3; 66,500 reticulocytes/μl, RI 3000–50,000) and thrombocytopenia (142,000 platelets/μl [RI 151,000–600,000]). A PCR panel testing for *Mycoplasma haemofelis* (MH), *Candidatus* Mycoplasma turicensis (CMt) and *Candidatus* Mycoplasma haemomintum (CMh) was submitted. The cat was administered orbifloxacin 3.4 mg/kg by mouth once daily pending results of the PCR panel and oral vitamin B supplementation (unknown type and dose) in the meantime.

The CBC 1 week later revealed improved normocytic and normochromic anemia and reticulocytosis (21% HCT and 94,600 reticulocytes/μl, respectively) and resolved thrombocytopenia (159,000 platelets/μl). The PCR panel was negative. The cat continued to receive orbifloxacin and vitamin B supplementation due to clinical improvement.

Over the following 2 weeks, the packed cell volume (PCV) ranged from 20.1–22.5% and reticulocyte count from 58,000–80,600 reticulocytes/μl. The cat also tested positive for feline immunodeficiency virus (FIV) and negative for feline leukemia virus (FeLV) on a lateral flow ELISA test kit (SNAP FIV/FeLV Combo) during this period of time. The cat was vaccinated for FIV 7 years prior to testing. One year prior to vaccination, the cat had tested negative for FIV through unknown diagnostic methods.

The cat presented to the VTH 1 week later. Abnormalities on physical examination consisted of a body condition score of 4/9 and a grade II/VI systolic parasternal heart murmur. CBC revealed a normocytic, normochromic, mildly regenerative moderate anemia (19.5% [RI 33.7–47.5%]; 83,500 reticulocytes/μl [RI 13,100–71,600 reticulocytes/μl]), marked thrombocytopenia (34,000 platelets/μl with platelet clumping; RI 149,000–532,000 platelets/μl), a neutrophilic left shift (185 bands/μl; RI 0–0 bands/μl) and lymphopenia (739 lymphocytes/μl; RI 804–9240 lymphocytes/μl) with reactive lymphocytes. Mild cardiomegaly was present on thoracic radiographs. Abdominal ultrasound identified mild splenomegaly and hypoechoic splenic parenchyma. Fine-needle aspirate cytology of the spleen demonstrated moderate macrophagic infiltration with phagocytosis of erythrocytes and erythroid progenitors and extramedullary hemato poiesis. Cytological evaluation of a bone marrow aspirate showed similar findings with moderately increased macrophages and phagocytosis of erythroid progenitors and erythrocytes. Erythroid hyperplasia was also noted. Rare phagocytosis of myeloid progenitors and megakaryocytic hyperplasia was also noted. The macrophages were similar in appearance to those observed in the spleen. The macrophages did not exceed 20% of the population. A bone marrow core biopsy was also collected, but a definitive diagnosis could not be obtained with the sample owing to low cellularity. Primary differentials at this point were hemophagocytic syndrome and hemophagocytic histiocytic sarcoma.^1–3^

In 2 weeks, the cat’s heart murmur progressed to a grade IV/VI parasternal systolic murmur. Splenomegaly was now identifiable on abdominal palpation. CBC revealed progression of anemia (15.6% HCT; 46,200 reticulocytes/μl), leukopenia (3500 white blood cells [WBCs]/μl; RI 4250–14,610 WBCs/μl) characterized by neutropenia (2065 neutrophils/μl; RI 2272–9639 neutrophils/μl) and a left shift (105 bands/μl) and mild thrombocytopenia (90,000 platelets/μl with manual estimate of 130,000 platelets/μl). Echocardiogram identified moderate, irregular, left ventricular hypertrophy with left atrial enlargement and left ventricular chamber enlargement, right ventricular chamber enlargement and right atrial enlargement. Small-volume pericardial effusion with pericardial thickening and pleural effusion were also noted. Primary differentials for these findings included either infiltrative disease of the heart secondary to infection,^4,5^ inflammatory disease or neoplasia,^6^ hypertension, or hypertrophic cardiomyopathy.^7^ Chronic anemia may also have played a role in the cat’s cardiomegaly as anemia has been associated with increased cardiac troponin 1 concentration, which is suggestive of cardiac myocyte damage.^8^ Blood pressure was not evaluated. Thoracic radiographs revealed progression of cardiomegaly but no evidence of congestive heart failure. Tests for *Histoplasma* species antigen in urine and serum IgM and IgG to *Toxoplasma gondii* were negative. Whole blood was tested using PCR for *Anaplasma phagocytophilum, Bartonella henselae, Bartonella clarridgeiae, Bartonella quintana, Ehrlichia* species, MH, CMh, CMt, *Rickettsia rickettsii* and *Rickettsia felis*, and was negative. In order to further differentiate between hemophagocytic syndrome and an underlying neoplastic condition, splenectomy was recommended.

Approximately 2 weeks later, splenectomy was performed without complication. The cat received buprenorphine 0.02 mg/kg IV q8–12h as needed for analgesia after surgery. Liver biopsies were collected at the time of surgery. Recheck echocardiogram prior to anesthesia revealed similar findings with progression of pericardial effusion and mild cardiac tamponade. Repeat thoracic radiographs did not reveal any evidence of pleural effusion or pulmonary edema, making congestive heart failure unlikely. The cat had type A blood and was given 20 ml blood-type-compatible packed red blood cells prior to surgery, which resulted in an increase in PCV from 18% to 21.5%. Postoperative PCV was stable at 23.5%. The cat was discharged and returned 1 week later for a recheck. The cat appeared more lethargic and the PCV had decreased to 15%.

**Figure 1:**
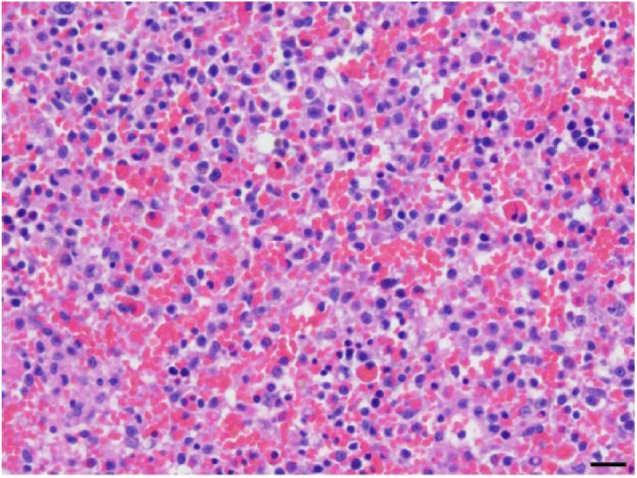
Spleen. Numerous mononuclear cells expand and efface the non-filtering areas of the splenic red pulp parenchyma. Occasional cells exhibit erythrophagocytosis. Hematoxylin and eosin. Scale bar = 20 μm

**Figure 2:**
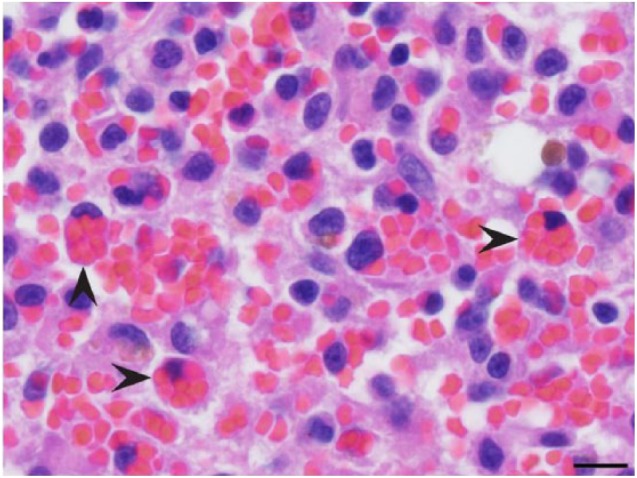
Spleen. Histiocytes exhibiting erythrophagocytosis (arrowheads). Hematoxylin and eosin. Scale bar = 10 μm

Histopathology of the spleen revealed multifocal aggregates of mononuclear cells with moderate amounts of eosinophilic cytoplasm, occasional variably sized intracytoplasmic vacuoles and distinct cell borders (Figure 1). Occasionally, macrophages within the aggregates were observed phagocytizing erythrocytes (Figure 2). There were also large numbers of megakaryocytes and both myeloid and erythroid precursors throughout the splenic parenchyma. Increased numbers of metarubricytes and rubricytes resulted in a myeloid to erythroid ratio of approximately 1:2. Multifocal regions of red pulp contained distinct sinuses void of erythrocytes. The hemophagocytosis within the spleen, paired with the extramedullary hematopoiesis, was suggestive of hemophagocytic syndrome. The macrophages did not exhibit characteristics of malignancy, which lowered the suspicion of hemophagocytic histiocytic sarcoma. The splenic histopathology was reviewed by five anatomic pathologists, who agreed with a diagnosis of non-neoplastic hemophagocytic histiocytosis. Histopathology of the liver was consistent with mild, multifocal extramedullary hematopoiesis. A diagnosis of hemophagocytic syndrome was made. PCR testing for FIV was performed on the splenic tissue and was found to be negative.

After splenectomy, there was no improvement in the anemia over the next 4 weeks, with PCV ranging from 15–17%. The cat was started on lomustine at 41.6 mg/ m^2^ orally every 3 weeks.^9^ The cat’s PCV fluctuated between 22% and 25% over the following 2 months. Evaluation with echocardiogram after the second lomustine treatment indicated stable cardiac disease. A grade 3 non-febrile neutropenia (864 neutrophils/μl) was encountered after the third treatment,^10^ so the treatment interval was extended to every 4 weeks. Lomustine was otherwise well tolerated.

Three months after initiating lomustine, disease progression characterized by thrombocytopenia, worsening anemia, ascites, pleural effusion, severe left atrial enlargement and static pericardial effusion was noted. There was concern that the cat may be in congestive heart failure owing to the presence of severe left atrial enlargement, pleural effusion and ascites. Abdominal fluid analysis was consistent with a protein-poor transudate. The cat was treated with vincristine 0.5 mg/m^2^ IV once and furosemide 1.5 mg/kg PO q12h. Over the following 6 days, a 25% reduction in body weight was noted, attributed to fluid loss and muscle wasting. The thrombocytopenia improved but the cat was considered too debilitated to receive any further chemotherapy. Owing to poor response to treatment, the cat was euthanized and no necropsy was performed.

## Discussion

Hemophagocytic syndrome, also called hemophagocytic lymphohistiocytosis (HLH), is a potentially life-threatening inflammatory condition characterized by inappropriate T-cell and macrophage proliferation and activation, which result in infiltrative disease and excessive cytokine production. In humans, macrophagic infiltration can occur in the liver, spleen, lymph nodes, bone marrow and central nervous system.^11^ These changes can result in organ system failure and uncontrolled phagocytosis of blood cells, often leading to severe cytopenias.^12^ In people, the syndrome is classified as a primary form, which is inherited, and a secondary or acquired form. The acquired form has also been termed ‘reactive HLH’ and occurs secondary to a number of conditions, including infection, malignancies, autoimmune disease and acquired immune deficiency.^11^

Diagnostic criteria for hemophagocytic syndrome have not been established for cats. The diagnosis was made in this case based on the presence of macrophagic infiltration in the spleen and bone marrow paired with hemophagocytosis, erythroid hyperplasia in the bone marrow, and extramedullary hematopoiesis in the liver and spleen. These changes resulted in anemia, thrombocytopenia and intermittent leukopenia. Diagnostic criteria previously used in a study evaluating 24 dogs with hemophagocytic syndrome included the presence of bicytopenia or pancytopenia in the blood and >2% hemophagocytic macrophages in bone marrow aspirates. They differentiated hemophagocytic syndrome from malignant histiocytosis by excluding patients with >30% macrophages in their bone marrow or if there were malignant features associated with the macrophages.^13^ Guidelines for hemophagocytic syndrome have been established in people, which include a known genetic defect and five of the following: fever, splenomegaly, bicytopenia or pancytopenia, hypertriglyceridemia and/or hypofibrinogenemia, elevated ferritin levels, elevated serum interleukin-2 receptor levels, decreased or absent natural killer cell activity, and hemophagocytosis in the bone marrow, cerebrospinal fluid or lymph nodes.^11^

Hemophagocytic syndrome is a rare condition in cats, and we are aware of only two other reported cases. The syndrome was diagnosed in one cat secondary to multiple myeloma,^1^ and another cat with hepatic lipidosis and suspected calicivirus infection.^2^ A definitive trigger for hemophagocytic syndrome in this case could not be determined. Hemophagocytic syndrome in people has been associated with HIV infection.^14^ Originally, there was concern the cat may have been infected with FIV because antibodies against FIV were detected. As PCR for FIV on the cat’s splenic tissue was negative, it was unlikely that the cat was truly infected. The cat in this case report was evaluated for other infectious disease, including histoplasmosis, toxoplasmosis, hematotropic mycoplasma and rickettsial disease. Histoplasmosis, toxoplasmosis and rickettsial disease have all been implicated in cases of reactive hemophagocytic syndrome in people.^15^ The cat reported here was not evaluated for feline calicivirus (FCV) because it was not displaying clinical signs consistent with FCV, such as sneezing, nasal congestion, conjunctivitis, gingivitis or oral ulceration. There is some limitation to this thought process as cats can live in a carrier state with FCV, but this cat, to our knowledge, had not displayed these signs historically.

It is difficult to determine if the cardiac changes observed in this case were associated with hemophagocytic syndrome. The ventricular wall thickening with irregular margins and thickened pericardium may have been secondary to macrophagic infiltration. Interestingly, hemophagocytic syndrome has been associated with a cardiomyopathy in people. Takotsubo-shaped cardiomyopathy, which is characterized by decreased contractility and apical ballooning of the left ventricle, has been described in people with hemophagocytic syndrome.^16^

There is no standardized treatment for hemophagocytic syndrome in veterinary medicine. People with hemophagocytic syndrome are commonly treated with etoposide and dexamethasone.^17^ This protocol is currently considered to be the standard of care for people.^18^ A similar protocol, which includes the addition of ciclosporin, has also been described.^19^ The goal of these protocols is to stabilize the disease before hematopoetic stem cell transplantation,^17–19^ which is not routinely available in veterinary medicine. In cats, etoposide is rarely used and the pharmacokinetics and pharmacodynamics are unknown. In this case, lomustine was selected as it is commonly used for histiocytic sarcoma and may improve survival in dogs.^9^ Lomustine has also been used in a case of feline disseminated histiocytic sarcoma.^20^

## Conclusions

To our knowledge, this is one of the few reports of hemophagocytic syndrome in a cat. This particular case may have been associated with a FIV infection and cardiac abnormalities. This is also the first case, to our knowledge, to describe treatment with lomustine for hemophagocytic syndrome in a cat.
